# Community and health worker perspectives on malaria in Meghalaya, India: Covering the last mile of elimination by 2030

**DOI:** 10.21203/rs.3.rs-3431734/v1

**Published:** 2023-10-16

**Authors:** Carinthia Balabet Nengnong, Mattimi Passah, Mark L. Wilson, Elisa Bellotti, Anne Kessler, Bibha R. Marak, Jane M. Carlton, Rajiv Sarkar, Sandra Albert

**Affiliations:** Indian Institute of Public Health Shillong; Indian Institute of Public Health Shillong; Indian Institute of Public Health Shillong; Indian Institute of Public Health Shillong

**Keywords:** Community health attitudes, KAP, malaria, infectious disease elimination, cultural practices, indigenous people, qualitative analysis

## Abstract

**Background:**

Malaria remains a public health problem in regions of northeastern India due to favourable bio-geographic transmission conditions, poor access to routine healthcare, and inadequate public health and healthcare infrastructure. This study was undertaken to better understand community members’ and health workers’ perceptions of malaria, as well as their knowledge, attitudes, and prevention practices related to the disease in Meghalaya state.

**Methods:**

The study included participants from three malaria endemic districts: West Khasi Hills, West Jaiñtia Hills, and South Garo Hills from 2019 to 2021. A total of 82 focus group discussions (FGD) with 694 community members and 63 in-depth interviews (IDI) with health personnel and traditional healers residing within the three districts were conducted. A thematic content analysis approach was employed, and NVivo12 software was utilized for data management.

**Results:**

Most participants reported a perceived reduction in malaria during recent years and attributed this to changing attitudes and behaviours in health seeking behaviour and effective government interventions. Local availability of testing and treatment, and an improved, more responsive health system contributed to changing attitudes. Long-lasting insecticidal nets (LLINs) were largely preferred over indoor residual spraying (IRS), as LLINs were perceived to be effective and more durable. Community members also reported using personal protective measures such as applying repellents, burning straw/egg trays, wearing long sleeve clothes, and applying ointments or oils to protect themselves from mosquito bites. While most participants acknowledged the role of mosquitoes in malaria transmission, other conditions that are not mosquito-borne were also attributed to mosquitoes by some participants. The communities surveyed have largely shifted from seeking traditional healers to using public facilities, although some participants reported switching between the two or using both simultaneously. Using the example of improved understanding of cerebral malaria which was previously attributed to mental illness due to ‘bad spirits’, participants explained how cultural and ritualistic practices had changed.

**Conclusions:**

Our findings reveal diverse perceptions among community members regarding malaria, its prevention, practices to prevent mosquito-transmitted diseases, and their opinions about the health system. A key finding was the shift in malaria treatment seeking preferences of community members from traditional healers to the public sector. This shift highlights the changing dynamics and increasing acceptance of modern healthcare practices for malaria treatment and prevention within tribal and/or indigenous communities. By recognizing these evolving attitudes, policymakers and healthcare providers can better tailor their interventions and communication strategies to more effectively address ongoing needs and concerns as India faces the ‘last mile’ in malaria elimination.

## Background

The WHO South-East Asia Region has made significant progress in reducing malaria incidence and deaths over the past two decades and accounted for ~ 2% of global malaria cases in 2021 (1). From ~ 23 million cases in 2000, the annual malaria incidence in the region decreased by 78% to approximately 5 million cases in 2020. Malaria deaths declined by ~ 75% during the same period (2). India is home to the vast majority of malaria cases in the region despite a decline from 20 million cases in 2000 to approximately 6 million cases in 2019 (3). Malaria remains a significant national health concern as India accounted for ~ 82% of the region’s malaria deaths in 2020 (2). In 2021, most malaria cases were caused by infection with *Plasmodium falciparum* though 40% were due to *Plasmodium vivax* (1). Ongoing prevention and treatment efforts targeting both species are required for continued progress in India (1).

India’s National Framework for Malaria Elimination (NFME) was established with the overall goals of malaria elimination (*i.e*., zero indigenous cases) by 2030 and maintenance of malaria–free status where malaria transmission has been interrupted through prevention of re-introduction. The framework has four objectives: (1) eliminate malaria from all 26 low (Category 1) and moderate (Category 2) transmission states/union territories (UTs) by 2022; (2) reduce annual incidence to < 1 case per 1000 population in all states and UTs by 2024; (3) interrupt indigenous transmission of *Plasmodium* throughout the entire country, including all high transmission states and union territories (UTs) (Category 3) by 2027; and (4) prevent the re-establishment of local transmission where it has been eliminated and maintain national malaria-free status by 2030 and beyond (4).

Most malaria cases in India are concentrated in the eastern and central parts of the country, particularly in states dominated by forested and hilly ecotypes and inhabited by tribal groups with poor access to health services. These states include Odisha, Chhattisgarh, Jharkhand, Madhya Pradesh, and Maharashtra, as well as the Northeastern (NE) states of Tripura, Meghalaya, and Mizoram. Transmission in these states is often persistent throughout the year due to various social and ecological factors. One of the key factors contributing to elevated transmission in these areas is the presence of large conflict-affected regions within the mentioned ecotypes. Further, there is often low community awareness about malaria prevention and control among the tribal populations residing in these regions. Lack of awareness and knowledge about preventive measures, such as the use of insecticide-treated bed nets, indoor residual spraying, and timely seeking of healthcare can altogether hinder efforts to reduce malaria transmission (4).

Analysis of Meghalaya data from India’s National Vector Borne Disease Control Programme (NVBDCP) indicated a general decline in malaria incidence during the past decade. (5). The observed decline may be attributed to several factors, including changes in treatment regimen (chloroquine replaced by artemisinin combination therapy), changes in land use and land cover in the study region, a rise in the proportion of subpatent, low parasitemia infections, and the first state-wide distribution of long-lasting insecticidal nets (LLINs) in 2016 (5, 6). In 2022, Meghalaya reported 463 malaria cases and 8 deaths, reflecting continued progress in malaria control efforts (7). Continued efforts to sustain and strengthen ongoing control, diagnostic, and treatment strategies will be crucial in further reducing the malaria burden in Meghalaya, and achieving the state and national goal of malaria elimination (5, 6). Meghalaya has reduced the malaria burden from Category 3 (Base year 2014) with an annual parasite index (API) > 1 to Category 1 in 2022 with an API of < 1(8).

To further support the Government of India’s NFME, this study was undertaken to investigate knowledge, attitudes, and practices regarding malaria and its prevention among community members living in rural, malaria-endemic areas of Meghalaya, in addition to documenting related perspectives of healthcare providers serving in the study villages and districts.

## Methods

### Study location and population

**Figure 1:**
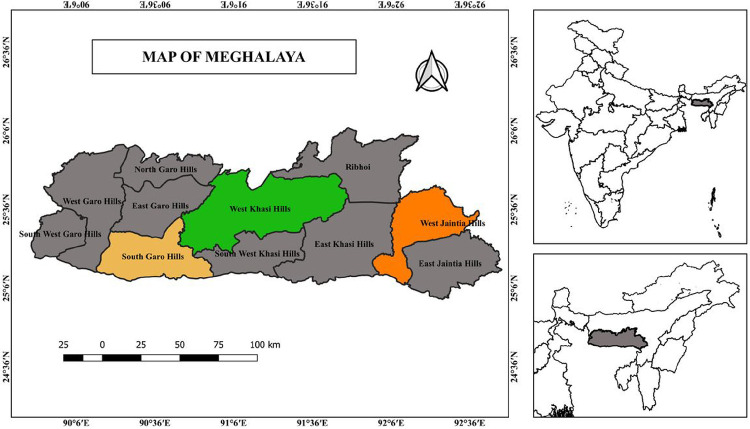
Map of Meghalaya with the study areas highlighted

Meghalaya is a largely forested, hilly state in the NE of India comprising 22,429 sq km with a predominantly indigenous or tribal population of ~ 3.81 million people (9). Meghalaya’s climate is influenced by the unpredictable nature of the monsoon, leading to significant variations based on altitude. This results in areas with delightful weather as well as those that encounter substantial rainfall (10). The study was conducted in three districts: West Khasi Hills (WKH), West Jaiñtia Hills (WJH), and South Garo Hills (SGH) (Fig. 1). These districts were chosen because they had the highest Annual Parasite Index (API) in Meghalaya between 2016 and 2019 (5).

### Data collection

Data were collected between August 2019 and November 2021 from residents of 23 villages served by four primary health centres (PHCs) across the three districts (Table 1). The villages were selected to ensure representation from each district and to cover a range of malaria prevalence within each district. In total, 82 focus group discussions (FGD) involving 694 community participants from the villages were completed. In depth interviews (IDI) were conducted with 63 service providers of which most were healthcare personnel serving the districts.

For IDIs, key health workers including Accredited Social Health Activists (ASHAs), Auxiliary Nurse and Midwife (ANM), nurses, Community Health Volsunteers (CHV), Medical Officers (MO), laboratory technicians, and pharmacists were interviewed. IDI were also conducted with the Anganwadi Workers (AWW), a focal point for implementation of initiatives under the Integrated Child Development Scheme (11), and with the traditional healers. When the perspectives of this group are presented collectively in this paper, the phrase ‘provider perspectives’ has been used interchangeably to represent the group.

With the exception of a select number of villages in the SGH, four FGD were conducted in each of the study villages with each group composed of 8–10 participants. Each FGD was comprised solely of either younger men (18–35 years old (yo)), younger women (18–35 yo), older men (≥ 36 yo), or older women (≥ 36 yo). Recruitment of participants was done with the help of the village headman, ASHA, and AWW.

Topic guides were developed for the FGD and IDI. The FGD topic guide focused on knowledge, beliefs, and attitudes that the community members had about malaria and its prevention. For interviews with healthcare personnel, the topic guide was adapted to align with the inquiries pertinent to a particular provider sub-group. For instance, for IDI with MO, the topic guide contained questions regarding current malaria treatment and diagnostic practices in the public health clinics.

Six trained research assistants conducted the field work. Preparatory visits were made by the team to each village to obtain permission from the relevant local authorities (e.g., village headman) before data collection was initiated. None of the village authorities that were approached refused to participate. Prior to each IDI or FGD, the participants were briefed about the purpose of the study, and written consent was obtained from each participant.

All IDIs and FGDs were recorded using a voice recorder (Sony ICD-UX570F). Each FGD was conducted by a two-member research team comprised of one facilitator and one note-taker. The facilitator led and conducted the FGD while the note-taker supported the facilitator and took detailed notes, including the participant’s seating arrangement and assigned number to identify the sources. The recordings were transcribed verbatim in the local language and subsequently translated to English by the same team. Quality checks were done through review by a third research team member. This helped determine the number of responses made by each participant for each question or probe and also facilitated coding in the analysis. Each FGD took from 25 to 90 minutes, while the IDIs ranged from 15 to 60 minutes.

### Approach to data analysis

During analysis, the same team members read and re-read each transcript to be familiar with the contents. Then, codes were developed and applied to the transcripts and reviewed again by other members of the team. Codes were grouped into categories or sub-themes and subsequently developed into broader analytical themes using a thematic content analysis approach. All qualitative data were managed and analysed using NVivo 12 software (Lumivero, Denver, Colorado). Transcripts were anonymised, and unique identifiers were given to each transcript or participant. The unique identifiers are provided at the end of quotes in the results section. For example R1_FGD_OM represents: R1, the participant number; FGD, focus group discussion; and OM, Older Men, the type of FGD. Similarly, IDI_MO_001 can be broken down as follows: IDI, in-depth interview; MO, designation of the respondent; and 001, IDI number.

## Results

The results are presented under six broad themes that are largely *a priori* and encompass participants’ perspectives on: (1) the prevalence of malaria, (2) their understanding of the causes of malaria, (3) their attitudes towards seeking healthcare for malaria (including traditional medicine), (4) their opinions on government interventions for malaria control, (5) their personal practices for preventing or managing malaria, and (6) the influence of cultural beliefs and perceptions of good health. The themes largely reflect community perspectives documented at the village level through FGD. Provider perspectives documented through IDI are presented briefly at the end of each section when relevant.

### Perspectives on malaria prevalence

This theme presents participant awareness of malaria disease burden and reasons for changes. Most participants in FGD across the three districts expressed that malaria incidence has declined over the years. Around 74 participants from WKH, 81 participants from WJH, and 98 participants from SGH verbally indicated a reduction in malaria cases. While many participants conveyed their thoughts verbally, others communicated non-verbally through actions such as nodding or shaking their heads. The numbers indicated in the results count only those who made explicit statements, which is often an underestimation of the numbers as it does not consider those who conveyed the message by non-verbal means or remained non-responsive.

In the FGD, the perceived reduction was attributed primarily to government interventions such as the distribution of LLINs and application of indoor residual spraying (IRS) as well as improved access to diagnostic and treatment facilities. These findings align with interviews with healthcare providers/personnel and traditional healers who reported similar improvements.

*“I just want to tell you that, 20 years earlier, I had also suffered from malaria. When one is infected with malaria, the palms and feet appear yellow in colour, and it takes time to get cured. When we go for a check-up in Shillong* (capital city)*, we get medicines, we take them, and then we get cured. But if we look at the generation now, malaria is not much of a problem as compared to the years before. Because now there are people here who do blood tests, provide us medicines, and also give us injections that can cure malaria. I feel that it is very accessible now. In our village now, very few people get malaria; they only get other kinds of infections.” – R2_FGD_OM_WKH*

While most FGD participants expressed that malaria had declined, a small number of participants reported that malaria is still a problem because of inadequate intervention measures available outside their homes, particularly in the agricultural fields. Participants further expressed that they are largely farmers by trade who spend considerable time outdoors, working in the fields or moving in forests or places with dense vegetation where they are frequently in contact with mosquitoes and are at a higher risk of contracting malaria.

‘It is really still a problem as we cannot protect ourselves from mosquito bites. If at home we use bed nets to protect ourselves from mosquitoes, when we go for our work in the forest, we roam around here and there…there are many mosquitoes that bites us…that might be infected and can cause malaria’ – R4_FGD_OM_WKH

Participants expressed that malaria is more prevalent in rural settings compared to urban areas where forests are less abundant. In the IDI, similar views on malaria prevalence were shared by providers. Both healthcare personnel in the public sector and traditional healers pointed out that malaria still persists in the area, and they believed that it could be attributed to the villages being surrounded by forests.

### Knowledge about malaria aetiology

This theme explored participants’ knowledge and understanding of the causes or origins of malaria, and it delved into their awareness of the role of mosquitoes as vectors, the transmission cycle, and other factors contributing to the spread of malaria. Most participants demonstrated awareness of how malaria is transmitted and could identify common signs and symptoms such as body aches, chills, fever, and sweating. Participants who had encountered malaria themselves or observed cases within their families were able to provide more detailed information about their experiences. About 70 participants from WKH, 73 from WJH, and 71 from SGH acknowledged that mosquitoes play a role in transmitting malaria. They believed that mosquito bites alone could cause malaria and that infected mosquitoes could transmit the disease to healthy individuals through subsequent bites.

The majority of participants were aware of one or more common signs and symptoms of malaria. The scope of symptoms reported included fever, shivering, headache, body ache, chills, joint pain, weakness, body swelling, tingling sensation, and flu like symptoms. The majority of participants stated that they would rely on blood tests to confirm a diagnosis of malaria; however, a small number of participants expressed the belief that they could identify malaria based on the signs and symptoms alone, by observation, without conducting any confirmatory test.

“The sign and symptoms that we usually see from a person who have malaria are fever, chills, weakness of the body, and also the tingling sensation….that’s what I know.”-R1_FGD_OM_WKH

In addition to observing the common signs and symptoms of fever and chills, participants reported that traditional healers also examine a person’s urine, eyes, and palms as part of their diagnostic method. The traditional healers that were interviewed explained that examining urine for changes in colour, consistency, or odour can be indicative of certain diseases or conditions, including malaria.

The majority of participants were unaware of diseases other than malaria that can be transmitted by mosquitoes. Participants occasionally categorised malaria with other conditions such as typhoid. When such views were expressed usually none of the other participants contested them. Some participants correctly mentioned other diseases that they believed could be spread by mosquitoes, such as Japanese Encephalitis and dengue fever. However, some conditions like diarrhoea and HIV/AIDS were incorrectly associated with mosquitoes. There were variations in the perceptions of mosquito-borne diseases among participants from different districts. For instance, younger men in SGH associated mosquitoes with skin infections and dengue fever, while younger women in the same district linked mosquitoes to stomach ache and joint pain.

“According to what I have heard malaria is the main disease spread by the mosquito bite but in my knowledge, I think typhoid might also be, and even diarrhoea because sometimes if we happen to consume the foods which are being infected by the mosquito, we can get typhoid and also diarrhoea.”-R1_FGD_OM_SGH

### Practices and attitudes towards seeking care for malaria

This theme presents participants’ reported behaviours and attitudes when it comes to seeking healthcare for malaria. Participant’s practices in terms of community preferences for treatment and utilizing healthcare services, such as visiting health facilities (and if so, public or private sector) or seeking care from traditional healers, were probed.

The majority of participants indicated they preferred seeking care for malaria from public healthcare institutions, such as PHCs, while a smaller number of participants reported a preference for seeking healthcare from private healthcare providers. Most participants reported having shifted their health-seeking behaviour from traditional healing practices to services at public health facilities. This transition is partly due to their belief that timely and effective treatment for malaria was available in the public health system. In the three districts, the participants and healthcare providers had expressed how the frontline health workers are now equipped with basic rapid diagnostic tests (RDT) for malaria and can promptly identify malaria cases, thereby ensuring more timely referral for appropriate treatment that improves outcomes and reduces the risk of complications.

**“** Before, when either me or any of my family members were being infected by malaria I will directly rush to the hospital or the PHC but now we can go to the ASHA because she also has the equipment which can run the test of malaria.” – R1_FGD_OM_WKH

Some participants described occasions where they received contradictory test results from different healthcare providers, including initial negative results from the ASHA. Such experiences contributed to reduced confidence in the testing equipment used by the ASHAs, and some participants said they would now prefer to go to the PHC directly for malaria testing.

“So…From my side I used to do the same as he had already told you, but there are chances that if we do the test with the ASHA it’s showing that there is no malaria detected but when we do the test in PHC it’s showing that malaria parasite is there in our body, so it was due to this reason that I never rely on ASHA test, I used to go directly to the PHC.” -R8_FGD_OM_WJH

In SGH, participants shared that it was problematic when it comes to visiting the health centre as it is located further from the village and there are no proper roads or public transportation to reach the facility. In keeping with this sentiment, the providers interviewed confirmed that villagers visit the health centre(s) with complaints of malaria, but the symptoms have sometimes subsided so they are unable to detect the parasite, especially when people self-medicate before seeking professional help. The providers indicated that this makes it difficult for doctors to accurately diagnose and appropriately treat the disease.

“We are living in rural areas, and people over here suffer a lot, especially due to financial constraints, and if we get malaria, we have to go to the doctor two to three times, and that too does not help cure the patient, and within two to three months there is relapse.”– R8_FGD_OM_WKH

While PHCs were mentioned as the preferred choice for most participants, health personnel did indicate that community members also visit traditional healers for treatment. Only a few participants from WKH and WJH stated that they would seek malaria treatment from traditional healers. The reasons offered for seeking traditional healers’ care included the perceived effectiveness of traditional healing methods, the limitations of modern medicine in certain scenarios, the (in) accessibility of healthcare services, and personal preferences. Traditional healers were often seen as providing quick care, and their services were said to be cheaper than those practicing modern medicine. The distance between someone’s village and the nearest PHC was also reported to influence the preference for traditional treatment since traditional healers are often located within or near the community, making them more easily accessible. This reduces the cost of travel and waiting time that would be associated with visiting a PHC.

*“I go wherever suits me because when I take allopathic* (modern) *medicine it is not effective. If I go to doctors, I’ll have to spend a lot of money in the medication. But when I go to traditional healers, the minimum charge will be hundred (Indian) rupees” – R10_FGD_YM_WJH*

Participants from WKH shared experiences of what they termed ‘yellow malaria’ for which they not only depended on traditional medicine but also sought confirmation and diagnosis through medical tests at PHCs. These participants also expressed a willingness to combine both traditional healing practices and modern medicine for treatment because they valued the confirmation and diagnosis from modern medical professionals and were less confident in diagnosis by traditional healers alone.

*“It* (“yellow malaria”) *is similar to malaria but it affects a person differently, the person looks pale and the skin turns yellow and there is swelling. This is the effect of yellow malaria and we take medicines from the traditional healers, there are times where it does not help then we go to the hospital but there are also times when it does not help even after the treatment from hospitals so we go to the traditional healers. We also have to take serious precautions and visit the doctor for any illness and not only depend on the traditional healers.” – R7_FGD_YM_WKH*

Some participants from WKH described that their primary reason for choosing traditional healers was a perceived lack of effectiveness of modern medicine. When individuals reported feeling that the medication prescribed by doctors was not helping them, they turned to traditional healing practices as an alternative solution. Further, there were instances reported where healthcare services were not readily available or accessible, such as when doctors were unavailable or when hospitals failed to respond adequately to the health needs of patients. In such situations, community members felt that they had no other option but to resort to traditional healing practices to address their health concerns. From the FGDs, the unavailability and inaccessibility of medical specialists contribute to the community members’ reliance on traditional healing practices, which represents their only viable choice for treatment.

“Yes…..we do go, because when we suffer from fever. Let’s say its Monday. The doctor will not be there. At night even if we come looking for help at the hospital we won’t be receiving any. So there are traditional healers we used to go who give local (Dawai Khasi) medicine…I had malaria in the past and had taken modern medicine from a medical practitioner and I did not get cured; so I tried from the traditional healers and from that time I drank medicines from them. I drank medicines for three times and was cured and I feel I received help from them.” – R6_FGD_YM_WKH

Participants from WJH shared their experiences of shifting between modern medicine and traditional medicine when they do not get cured. In cases where modern medicine fails to provide their expected outcome(s), they turn to traditional medicine as an alternative. Participants from WJH reported using both traditional and modern medicines to treat malaria. They believe that using both types of medicine provides them with benefits, and some participants reported that they saw no reason to not use traditional medicine alongside modern medicine.

“Yes, we also take herbal medicines as it does not have any side effects and cause no harm if we take along with modern medicine” – R2_FGD_YM_WJH

In contrast participants from SGH district reported that community members have largely stopped seeking traditional healers’ help for malaria treatment as their awareness of diagnostic tests, treatments, and services in PHCs or hospitals has increased. The availability of diagnostic tools, such as those used by the village ASHA and ANM in the health sub-centres, has contributed to the villagers’ preference for seeking help from these healthcare providers.

Traditional healers from WJH and SGH shared their traditional practices for diagnosing and treating malaria. Briefly, they employ various methods of preparing medicines, use different types of remedies, and assess the perceived effectiveness based on the reported experiences of clients. A traditional healer from WJH described a practice specifically for treating malaria that has been passed down through generations in his family whereby he infuses a thread spun from fibres of a particular plant with oil, turmeric and garlic that the patient is instructed to wear.

A traditional healer from SGH provided insights into his practice and the preparation of his medicines according to the patient’s specific ailment. He said gathering the required herbs is often time-consuming, as he needs a full day of travel to the forest to collect them. The healer acknowledged that not all patients will be cured by his treatments. He explained that the effectiveness of certain herbs depends on the individual’s body, and that different patients may have different preferences for treatment, including seeking assistance from other traditional healers or opting for modern medicine. His treatment involves mixing herbs with warm water and instructing patients to drink the mixture. For headaches, he recommends the use of “*Poron*” which refers to ground herbs that are used as a poultice on the patient’s head.

### Attitudes towards government implemented malaria control interventions

This theme explored participants’ perceptions and attitudes on the malaria control interventions implemented by the government (e.g., IRS and LLIN) and assessed their opinions on the effectiveness, accessibility, and overall quality of these interventions. Overall, the community members acknowledged a lower acceptance of IRS compared to LLINs which appeared to be attributable to perceived ineffectiveness and inconvenience of IRS.

About 601 participants across the districts felt that IRS acceptance was low. Key reasons that participants shared for low acceptance of IRS was the perceived temporary nature, as effects diminish over time, requiring repeated applications. Pregnant women were concerned about the potential effects of the insecticide on their health and the health of their unborn child. It was reported that the IRS solution had a strong odour and caused dizziness, especially for pregnant women and children. Some community members perceived IRS as making their houses dirty due to the residue left behind by the insecticide. Community members expressed feelings that the IRS solution was not prepared well and of the possibility that excess water was added, thus reducing its effectiveness.

Most participants across the districts were of the viewpoint that LLINs are more effective compared to IRS. This aligned with interviews with healthcare providers who also highlighted the benefits of government-distributed LLINs for the community members. Specifically, they mentioned that most villagers face financial constraints and cannot afford to purchase bed nets, particularly if they have large families. Further, LLINs were stated to be helpful during the summer season when individuals tend to sleep without covering themselves with blankets or shawls. Some of the participants indicated that bed nets help to protect not only from mosquitoes but also from other insects.

*“Yes, what I believe is that the use of bed nets was of prime importance. Since they do not agree to spray on the areas that we want, it* (IRS) *is useless because they want to spray indoors when there are more mosquitoes around the pig sty….” – R3_FGD_OW_WJH*

A few of the participants expressed that the number of bed nets provided to them was insufficient for big families. Affordability was also cited as a major challenge by the participants as well as healthcare providers, as families found it difficult to purchase extra bed nets beyond what the government supplied free of charge.

### Personal malaria prevention practices

This theme focused on the individual behaviours and practices adopted by participants to prevent malaria both inside and outside their homes, as well as when they are working in their agricultural fields. It examines the range of preventive measures individuals employ to protect themselves from malaria. Participants reported that they use bed nets, mosquito repellents like coils, vaporisers and creams inside their homes, and they keep their windows and doors closed in the evening to help minimize the entry of mosquitoes into the house. Some participants also described keeping the veranda lights on, as they believe it reduces mosquito entry into the house.

“The first prevention measure is bed nets, the second is using smoke or repellents and the third is to leave the light open but in the fields, nothing can be done in the end the mosquito gets the advantage.” – R10_FGD_YM_WJH

Participants reported implementing personal preventive practices to reduce the risk of malaria transmission, especially outside their homes where there were fewer preventive options. Community members described burning leaves, straw, wood, egg trays, jute bags, and carton boxes outside their homes. They also reported boiling water before drinking it, and taking action to ensure proper drainage around their homes to prevent the accumulation of stagnant water.

The farmers involved in *Jhum* cultivation and forest dwelling participants reported increased risk of malaria due to their occupational and living conditions. Some participants also indicated that they would wear long overalls and apply oil when working in agricultural fields to prevent mosquito bites. However, some participants expressed concerns about the effectiveness of these personal protection measures.

“As farmers we cannot escape our work and will have to work day and night, we will have to bear mosquitoes bite that is the main reason we become ill.” – R10_FGD_YM_WKH

Additionally, some community members practiced self-treatment using readily available medications such as paracetamol, which can be obtained from local shops without a prescription. Participants shared that they practiced home remedies and self-medication whenever they started to experience symptoms of malaria because it is less expensive and easily available. Participants shared that they prefer self-medication as it saves travel time to the health centre and avoids losing time at work, as most are daily labourers.

Other practices reported include using steam inhalation from lemon leaves, bamboo leaves, and yam stems; drinking lemon water with honey for symptom relief; and applying oil or honey on the body to keep warm during chill episodes.

### Cultural beliefs and perceptions of good health

During the FGDs, participants provided an overview of their understanding and perceptions of what they considered to be ‘good health.’ Many participants started with comments on physical health and the absence of illness but then went on to include a more holistic state of being that included various aspects of a person’s life. As the conversations progressed, some participants delved into the cultural beliefs and practices related to health that prevail in their communities.

Participants shared how they and their fellow community members relied on local beliefs and performed specific rituals to treat health ailments. They explained, however, that attitudes and practices towards malaria have changed over the past decade. For example, there was a time when villagers did not know about cerebral malaria (referred to as ‘brain disease’ in WKH), and assumed that it was mental illness caused by evil spirits. After seeking bedical diagnosis and treatment from healthcare professionals, they accepted that this was a more severe type of malaria with the same underlying cause. With the passage of time and changes in the community’s attitude and healthcare seeking behaviour, certain cultural practices regarding malaria have also changed. Of note, some community members still seek help from traditional healers depending on the ailment, even though most of the community members have largely shifted to modern medicine for treatment of malaria.

Some participants, particularly older men, mentioned rituals that were performed by their ancestors. One such ritual involved sacrificing a rooster and using eggs for health prediction(s). These rituals were believed to have a connection with health and were performed as a means of understanding and addressing illnesses, including malaria.

*“When we talk about the disease, there are many types of diseases because there is this new disease that has the same symptoms as malaria, but this new disease “ngi phet biej”* (literal translation in English could mean being in the wrong state of mind), *so that’s why we need to go to the doctor. Many of the people out here, when they have this fever and cold, they usually “kren biej”* (speak nonsense)*, so they thought it was due to witchcraft done by someone, because this type of disease affects the brain. But when they visited the doctor, they said... It is a disease of the brain. There are many people who get this brain disease. Oh… I remember it’s the brain malaria that the doctor mentioned...” – R8_FGD_OM_WKH*

Participants and respondents placed significant emphasis on physical well-being as a primary indicator of health. Overall, their perspectives on physical health encompassed aspects such as physical fitness, freedom from illness, cleanliness, healthy habits, and socioeconomic implications. The participants considered physical fitness and freedom from illness as essential attributes of a healthy individual. They associated good health with the ability to perform daily tasks effectively, suggesting a positive correlation between physical well-being and productivity.

“Well Kong [common local name often referring to a woman], according to me if a person does not exercise or do any physical activity he/she will not be healthy. Even if they do not eat good food or maintain a good diet they will not be healthy” –R2_FGD_YW_WJH

The participant discussions emphasized the value of cleanliness, both at the household and locality levels, in maintaining good health. Clean surroundings and personal hygiene were viewed as essential factors for preventing diseases and promoting well-being. Many participants acknowledged the importance of healthy habits such as good diet, getting proper sleep, taking regular rest, and engaging in physical exercise. These behaviours were seen as crucial for preventing the need for frequent visits to healthcare facilities and expenditures on medication. The community members recognized a link between physical health and socioeconomic well-being. A healthy person was perceived as someone who could work, earn a living, and fulfil their familial responsibilities.

“Health is maintaining cleanliness, keeping our body clean, keeping our surroundings clean”. -R3_FGD_Women_SGH

The participants also spoke of the connections and interactions that individuals have with each other and with their communities, and the importance of social exchange and community involvement for overall well-being. Discussions included emphasis on the importance of the quality of relationships, social support networks, and the sense of belonging. The FGDs reflected participants’ understanding of the socially interconnected nature of health. By acknowledging the social aspects of health, the participants highlighted the role of community and social relationships in promoting and maintaining overall health and enhancing their quality of life. The participants emphasized that socializing and engaging with others in the community positively impacts a person’s well-being. Examples of community support and helping one another were shared, highlighting the mutual assistance and sense of belonging that contribute to well-being. Some participants explicitly recognized the interrelationships between physical, social, and mental aspects of health. They understood and articulated that when a person is physically fit and free from stress or tension, their ability to interact effectively within the community is enhanced. Good health was seen and explained as a foundation for maintaining communication and relationships with family, friends, and neighbours. Moreover, the participants acknowledged that being healthy enables individuals to actively contribute to society.

“We also need to have a good relation with our family members, neighbours, and friends because if we don’t get along with our friends, neighbours and families then we don’t feel good and our mind is also not at ease. So to have a good health we need to be aware and take care of ourselves” - IDI_CHV_051_SGH

Mental health, which involves an individual’s psychological and emotional well-being, was recognized by some participants as affecting physical health, social relationships, and community engagement. The participants acknowledged that mental health has a profound influence in every aspect of a person’s life and recognized that mental well-being can affect physical abilities, social interactions, and the overall functioning of a person. The discussions highlighted the participants’ understanding that an individual’s ability to engage in social relationships and community life can be disrupted if they do not have a clear and healthy mind. The participants expressed their belief that meeting people every day and engaging in social interactions is beneficial for mental health. They recognized the positive impact of socialization and specifically indicated that it provides opportunities for connection, support, and the exchange of ideas.

“Health is very important because we usually think that health is about being free from diseases but is also about having a healthy mind which is also very important.” R1_FGD_OW_WJH

## Discussion

This study provides insights into how tribal people from three districts of rural Meghalaya understand, perceive, treat, and prevent malaria. Using the insights gathered from community members and local health workers, interventions can be tailored to address specific needs and challenges faced by these communities through the crafting of targeted interventions and improvement of existing strategies and programs. The insights gathered can also aid the development of community engagement strategies and health education campaigns to increase awareness about malaria prevention methods, such as the use of LLINs, acceptance of IRS, and timely seeking of medical care (12). There were notable differences in bed net usage and related practices when compared to a study from Odisha state (India), which reported low use of bed nets with the utilization being determined by seasonality, affordability and alternate uses of nets (12). Also, in terms of health-seeking behaviour, the Odisha study reported that community members relied considerably on unqualified and traditional healers, with low trust in community health workers.

The majority of participants were forest dwellers/farmers who shared that they considered malaria to be much less of a health problem than in previous years. This was consistent with another recent study involving slash and burn cultivators in Bangladesh (13), which borders Meghalaya state to the south. This perception among Meghalaya tribal people conforms to published reports from surveillance and clinic data that document a dramatic decline in malaria incidence and deaths over the past few years (5). Not only did the study participants accurately report perceptions of declining malaria, but most community members also possessed accurate information regarding the causes and prevention of malaria. Such knowledge is valuable for the acceptance and proper utilization of contemporary malaria prevention measures (*e.g*., LLIN, IRS) and may reflect the government’s efforts to expand use of these measures and inform people of their value and benefit. Many participants in all three districts surveyed were also able to correctly identify common signs and symptoms of malaria. These results align with those from other investigations conducted in Madhya Pradesh state in India and Bangladesh, which also reported that local residents possessed accurate knowledge about malaria and were able to identify the common symptoms (13, 14).

Most of the Meghalaya study participants preferred to visit government or private health centres for malaria treatment. This is consistent with a recent study conducted in Jammu and Kashmir where nearly all participants expressed a preference for being treated at government healthcare facilities (15). The findings of this study suggest that members of tribal communities increasingly recognize the value and effectiveness of malaria treatment at modern (allopathic) healthcare facilities where care is provided by trained medical professionals. A few participants from two districts (WKH, WJH) still expressed a preference for traditional healing practices, which was based on their personal beliefs or cultural traditions. They indicated a pluralistic health seeking behaviour that switches from one system to the other based on perceived efficacy or even use of both simultaneously. This possibly reflects the community members’ pursuit of effective and alternative solutions, as well as a belief in the potential benefits of different types of healthcare. But participants from SGH district, the district with the largest number of malaria cases currently in Meghalaya, have largely switched from seeking traditional medicine to modern medicine for malaria. This is notably different from a cross sectional survey on medical pluralism in Meghalaya that reported high awareness and wide use of both modern and tribal traditional medicine (16).

In this study, traditional healers across the districts shared that they would refer cases of malaria to other healers or healthcare facilities if patients did not respond to their treatments. This is similar to what was reported in a study of traditional healers in Tanzania, where most traditional healers indicated they used a system for referral to other healers or health facilities for severe cases or when their treatments were ineffective (17). Similarly, participants have indicated their flexible approach to seeking medical care, where they would visit either traditional healers or medical doctors based on the specific ailments they were experiencing. This is similar to the finding from an earlier study conducted among traditional healers in Meghalaya, which highlighted the utilization of both types of services depending on the nature of the illness. (18)

Study participants reported reliance on frontline health workers for confirmation of malaria through blood testing. Similar findings were reported in other studies conducted in Bangladesh and India where community health workers were identified as the initial point of contact for suspected malaria cases (13–15). Whether they assist with diagnosis and/or treatment, frontline health workers who often also reside in the community play a crucial role in malaria diagnosis and treatment, and ultimately in reducing transmission.

The majority of participants in all three districts reported LLINs and IRS as the main interventions that they used to prevent malaria. Most participants also reported that LLINs are effective and preferred them over IRS, which is consistent with studies conducted in Meghalaya that found low acceptance of IRS (19, 20). These findings contrast with those reported in Madhya Pradesh, India where a high percentage of households indicated acceptance of IRS to prevent malaria (21). Although LLINs were valued and used by participants in the present investigation, a key challenge indicated by a few participants was that government-provided LLINs were insufficient in providing coverage for the entire family. Participants reported financial constraints in acquiring additional bed nets, particularly in households with larger family sizes. This was also found in the study conducted in Odisha state, India which revealed that irregular use of bed nets was due to few nets and high net cost, especially in households with a large family and diverse sleeping patterns (22). Nevertheless, sleeping under bed nets was identified as the most common preventive measure adopted by the community members which aligns with results from the Bangladesh study (13). Taken together, these findings emphasize the importance of addressing the financial and logistic barriers to bed net access, especially for households with limited resources and larger families.

Use of additional, personal prevention methods to reduce malaria risk further illustrated the concerns and practices of people in the region. Participants shared that they would burn dry neem tree (*Azadirachta indica*) leaves to produce smoke that would repel mosquitoes, especially when they were out in the fields. This practice was also reported by community members in Madhya Pradesh, India (14). Similarly, the burning of insecticide-containing repellent coils to ward off mosquitoes was also reported by some community members, much like the technique used by forest dwellers in the Bangladesh study (13). Community members also reported engaging in practices such as cleaning their surroundings and eliminating stagnant water to prevent mosquito breeding reservoirs outside their homes. These practices are similar to those reported in both Bangladesh and Côte d’Ivoire (13, 23).

The malaria diagnostic method reported by traditional healers in our study involved observing the colour of the palm, eyes, and urine. In Côte d’Ivoire, the concept of “*ewuego*”, the local folk term to describe a deep yellow colouring of these body parts, similarly suggests that colour change is associated with the presence of malaria (23). Other parallels of note between our findings and those of the traditional healer study in Côte d’Ivoire include higher reports of observing the symptoms associated with malaria in the summer season and a community awareness of drugs to treat malaria (*i.e*., chloroquine) and manage their symptoms (*i.e*., paracetamol / acetaminophen) (23).

One limitation of this study was that FGD participant recruitment was facilitated through the headmen and frontline health workers in each village, which may have favoured or excluded certain individuals or groups. The different power dynamics within some discussion groups could have intimidated certain individuals and prevented them from expressing their thoughts freely during the FGD, although the research team made efforts to encourage every member to voice their opinions in each FGD.

**Figure 2:**
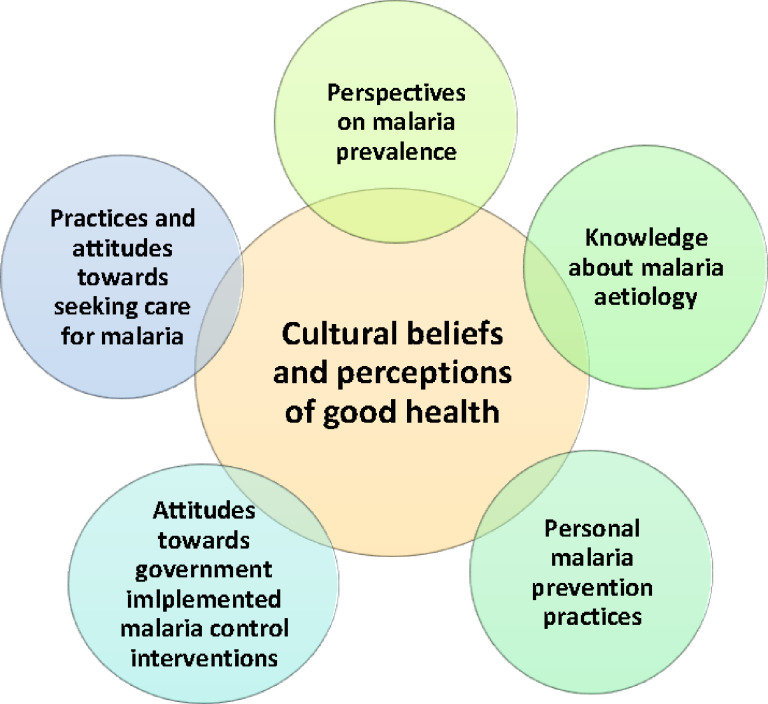
Conceptual map of the themes that emerged from the analyses

A framework showing the interconnected and dynamic nature of the themes from this study of tribal villagers living in rural villages of Meghalaya is shown in Fig. 2. People’s perspectives on malaria prevalence, knowledge about malaria causes, personal prevention practices, attitudes toward government prevention programs, and treatment-seeking practices represent critical cultural beliefs and practices and are important to consider as knowledge of malaria changes. Indeed, since the Government of India aims to eliminate malaria by 2030 (24), this study highlights some challenges and opportunities for attaining this goal. The momentum achieved in reducing malaria can be enhanced by utilizing local understanding about malaria aetiology, treatment, and prevention, and elevate existing strategies or develop new ones for “last mile coverage” in remote areas. While considerable knowledge about malaria existed in the communities studied, there were also misconceptions and a tendency to conflate malaria with conditions involving other pathogens and vectors. Such misconceptions may not have immediate negative consequences in the community but should be addressed to mitigate misunderstandings and promote transmission and disease prevention. Since people continue to trust and utilize the services of traditional healers in some districts, identifying methods for the formal health sector to engage with the traditional practitioners in the informal sector will be required as the country moves toward national elimination.

## Figures and Tables

**Table 1:** PHC areas sampled and the number of FGDs and IDIs undertaken in villages of three districts of Meghalaya, India.

Focus group discussions (FGD)					
District	PHC area	No. of FGD villages	No. of FGDs	No. of FGD participants	No. of IDI responders
**West Khasi Hills (WKH)**	Nonglang	6	24	199	17
**West Jaifitia Hills (WJH)**	Nartiang	3	12	116	16
	Barato	3	12	100	11
**South Garo Hills (SGH)**	Siju	11	34	279	19
**Total**	**4**	**23**	**82**	**694**	**63**

## Data Availability

All data supporting our findings are contained in the paper. Data gathered from the transcripts can be made available from the corresponding authors upon reasonable request.
